# Theory-guided design of high-entropy alloys with enhanced strength-ductility synergy

**DOI:** 10.1038/s41467-023-38111-6

**Published:** 2023-05-02

**Authors:** Zongrui Pei, Shiteng Zhao, Martin Detrois, Paul D. Jablonski, Jeffrey A. Hawk, David E. Alman, Mark Asta, Andrew M. Minor, Michael C. Gao

**Affiliations:** 1 National Energy Technology Laboratory, Albany, OR 97321 USA; 2 ORISE, 100 ORAU Way, Oak Ridge, TN 37830 USA; 3 Department of Materials Science and Engineering, University of California, Berkeley, CA 94720 USA; 4 National Center for Electron Microscopy, Molecular Foundry, Lawrence Berkeley National Laboratory, Berkeley, CA 94720 USA; 5 Materials Sciences Division, Lawrence Berkeley National Laboratory, Berkeley, CA 94720 USA; 6 Present Address: School of Materials Science and Engineering, Beihang University, Beijing, 100191 China; 7 Present Address: Tianmushan Laboratory, Xixi Octagon City, Yuhang District, Hangzhou, 310023 China

**Keywords:** Mechanical properties, Metals and alloys

## Abstract

Metallic alloys have played essential roles in human civilization due to their balanced strength and ductility. Metastable phases and twins have been introduced to overcome the strength-ductility tradeoff in face-centered cubic (FCC) high-entropy alloys (HEAs). However, there is still a lack of quantifiable mechanisms to predict good combinations of the two mechanical properties. Here we propose a possible mechanism based on the parameter κ, the ratio of short-ranged interactions between closed-pack planes. It promotes the formation of various nanoscale stacking sequences and enhances the work-hardening ability of the alloys. Guided by the theory, we successfully designed HEAs with enhanced strength and ductility compared with other extensively studied CoCrNi-based systems. Our results not only offer a physical picture of the strengthening effects but can also be used as a practical design principle to enhance the strength-ductility synergy in HEAs.

## Abstract (web summary)

There is a lack of guiding parameters to design metallic materials such as high-entropy alloys with strength-ductility synergy. Here, the authors propose such an effective parameter κ, the ratio of short-ranged interactions between closed-pack planes, experimentally validated by six alloys.

## Introduction

**Fig. 1:**
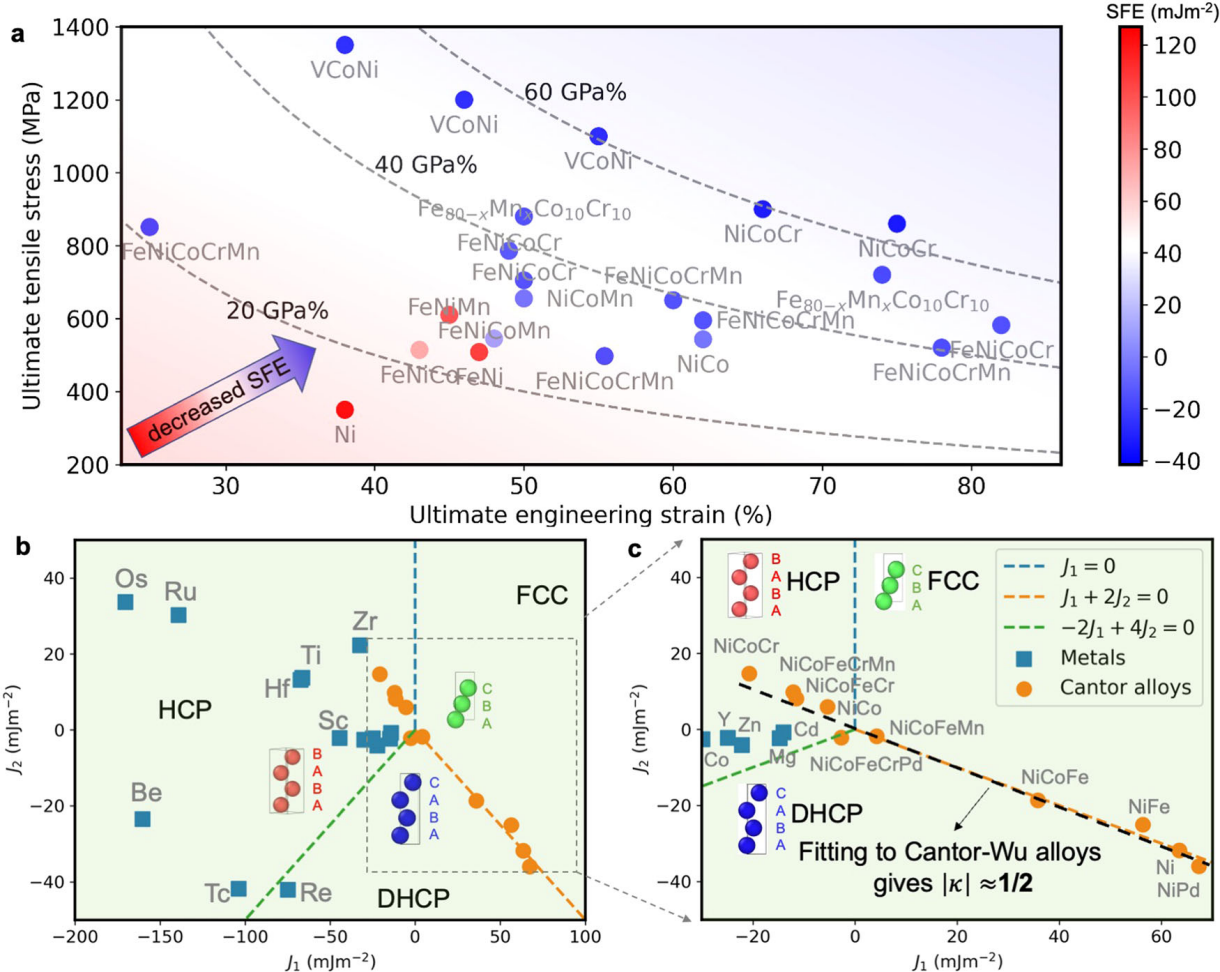
Materials of close-packed structures in the *J*-parameter space and their mechanical properties. **a** The room temperature mechanical properties for representative concentrated alloys as a function of zero-temperature SFEs. Decreased SFEs give better work hardening for these concentrated solid solutions since their tensile-test curves are similar in shape. The dashed lines are used to guide the eyes, which indicate the combined mechanical performance of ultimate engineering strain (ductility) and ultimate tensile stress (strength). **b**, **c** Materials of close-packed structures in the two-dimensional space of the ANNNI model. All materials are shown in **b**, and panel **c** is a magnified view of the part enclosed by the dashed line in panel **b**. The materials include 14 pure metals^22^, the extensively studied Cantor alloy and its subsystems^6^. The error bars (standard deviations) for SFEs and $J$ parameters are not shown here since they are not actual error bars but a physical property inversely proportional to the square root of system size when enough configurations are considered in calculations (see supplementary material). The experimental data for concentrated alloys include [a]-Ref. ^6^; [b]-Ref. ^40^;[c]-Ref. ^41^;[d]-Ref. ^30^;[e]-Ref. ^42^. Tabulated data can be found in Table S2.

The FCC high-entropy alloys (HEA) have attractive mechanical properties^1–4^, such as yield stress, ductility (plastic strain to fracture), damage tolerance, etc.^1,3–5^ Representative examples of such alloys include the Cantor alloy CoCrFeMnNi and some of its subsystems (i.e., the Cantor-Wu alloys)^6^. While the high yield stresses of these materials have been explained and predicted by theoretical models^7,8^, efforts are still needed to establish accurate micro-scale parameters or models to quantitatively or semi-quantitatively describe the excellent toughness. Gludovatz et al. found that the Cantor alloy possessed the highest toughness recorded at cryonic temperatures. In addition, its ductility improves upon cooling, which is different from most alloys^3^. This phenomenon has been qualitatively explained by mechanical nanotwinning that can be grouped into the twinning-induced plasticity (TWIP) effect. Another typical example of gaining an improved toughness is introducing a second phase assisted by the transformation-induced plasticity (TRIP) effect^4^. The TWIP and TRIP mechanisms are usually considered to be controlled by the intrinsic stacking fault energy (SFE)^9^. In HEAs, particularly those with excellent strength-ductility combinations (e.g., CoCrFeNiMn, CoCrFeNi, and CoCrNi), extremely low to negative SFEs have been identified^10–13^. Nonetheless, an excellent combination of strength and ductility is too complicated to be accurately described by a single parameter. To better characterize it, we introduce an independent parameter associated with close-packed structures (CPSs). The CPSs are sequences of A, B, and C layers (see Fig. 1b)^14,15^. Various CPSs have been reported in alloys like CoCrNi^11,16–18^. Like TWIP and TRIP, the CPSs can also induce plasticity to enhance ductility and strength. Both TWIP and TRIP depend on unique and clearly defined CPSs: twin stackings for TWIP and HCP embedded in the FCC matrix for TRIP. Following the convention, we coined the term generalized TWIP mechanism to describe a possible mechanism that depends on multiple CPSs rather than one CPS. The behavior of planar defects depends on the interplanar interactions. Materials with similar interplanar interactions can have different SFEs, which will become more apparent later when we introduce a mathematical expression. SFE proportional to the free energies of the FCC and HCP structures^15^ is not an adequate descriptor. For example, when CPSs are energetically degenerate, there is no strong formation preference between CPSs thermodynamically, such as twins and HCP. Therefore, additional parameters are needed to describe their behavior.

Here, we identify a degree of freedom in addition to SFE, i.e., the κ parameter, to characterize the proposed physical mechanism. The parameter has practical applications in designing materials. Guided by it, we synthesize HEAs showing excellent ductility and high strength at room temperature. This provides an insight that it is not just the SFE but, more generally, the interactions between stacking faults that is important for designing to balance strength and ductility.

## Results

### Metals and high-entropy alloys with close-packed structures

Materials with CPSs are ubiquitous in nature^19,20^. About two-thirds of the pure elements exist in CPSs, primarily consisting of FCC, hexagonal close-packed (HCP), and the less common double HCP (DHCP) structures. Ising models have been employed to describe broad physical phenomena, from magnetism to chemical ordering. One of their special forms, i.e., the Axial-Next-Nearest-Neighbor-Ising (ANNNI) model^21^, has been successfully used to study the typical CPSs (e.g., FCC and stacking faults) in pure metals^14^ and conventional (dilute) alloys^15,22^. The ANNNI model describes the enthalpy $H$ of one system in the form of $H={{NJ}}_{0}-{\sum }_{i}{\sum }_{n=1}^{2}\,{J}_{n}{S}_{i}{S}_{i+n}$, where ${S}_{i}$ is a spin-like discrete variable determined by the stacking order of close-packed plane *i* and *i* + *1*. If a plane and its next neighboring plane follow the order in FCC stacking, ${S}_{i}$ is 1; otherwise, ${S}_{i}$ is −1. $N$ is the number of planes, and ${J}_{n}$ is the interaction parameter between ${S}_{i}$ and its *n*th-nearest neighbors (*n* = 1, 2)^15^. More details are referred to the method part and the supplementary material.

The ultimate tensile stress and strain are shown in Fig. 1a for alloys in close-packed structures. Each data point is colored by the alloy’s zero-temperature SFEs calculated by density functional theory (DFT, see the “Methods” part). The high-strength and high-ductility FCC HEAs have extremely low to negative SFEs, consistent with the classical one-dimensional SFE picture in alloys like steels. Typical HEA examples include the Cantor-Wu alloys^2,6^. We perform DFT calculations to obtain the total energies for the metals and alloys and parameterize the energies according to the ANNNI model. This operation yields the values of $J$ parameters (${J}_{1}$ and ${J}_{2}$) for a 2D plot where each alloy occupies a unique position. The whole plot is shown in Fig. 1b, and a crucial part of it is magnified in Fig. 1c. Finally, the fitted line is represented in Fig. 1c. Surprisingly, when these alloys are arranged in the $J$ parameter space, they follow a straight line, a phenomenon that has never been reported before.

**Fig. 2:**
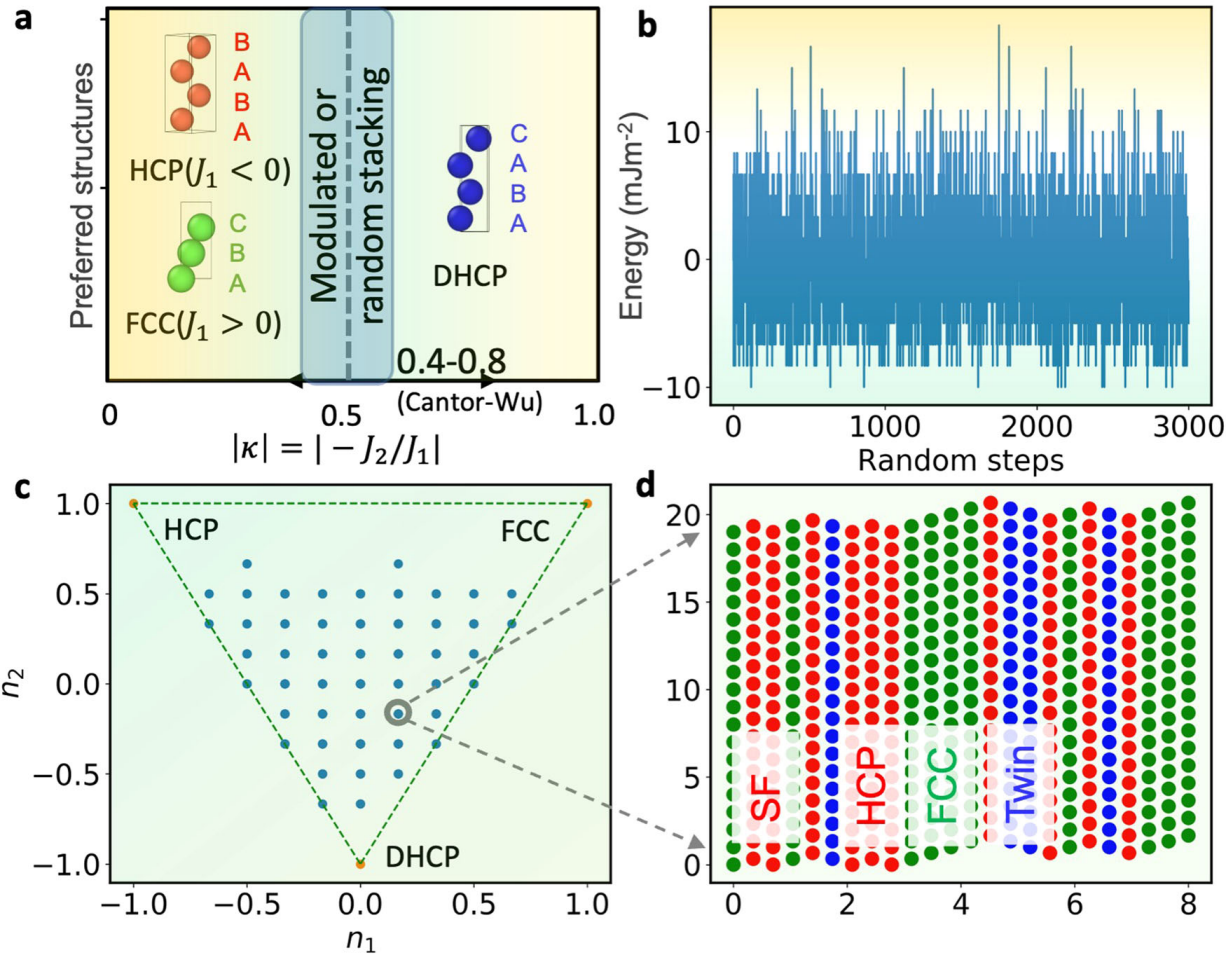
The schematic phase diagram of close-packed structures by the ANNNI model. As a representative system, a model is adopted with parameter values of a typical Cantor-Wu alloy ($\kappa=-$½, ${J}_{1}=-20$ mJm^−2^, ${J}_{2}=10$ mJm^−2^). **a** The phase diagram for the three-dimensional ANNNI model in (${k}_{B}T/|{A}_{0}{J}_{1}|$, $\kappa$) space (reproduced from ref. ^10^). At low temperatures, FCC and HCP structures share the space $\kappa <$½, and the switch between the structures depends on the sign of ${J}_{1}$. DHCP always occupies the space $\kappa >$½ below the critical temperature. The multicomponent Cantor-Wu alloys ^66^ have $|\kappa|\in$ [0.4,0.8]. A model system with *κ* = ½ is taken as an example for the random simulation of close-packed stacking. **b** The energies of different configurations with a period up to 24 layers with 3000 steps. **c** The different configurations find their positions in the phase space of (${n}_{1}$, ${n}_{2}$). The three phases: FCC (1,1), HCP (−1,1), DHCP (0, −1). All CPSs can be reduced to a combination of the three fundamental units (FCC, HCP, DHCP). **d** The schematic for the nanoscale stacking sequences of point (${n}_{1}$, ${n}_{2}$) = (1*/*6, −1*/*6). Green layers are FCC stacking, blue ones for twin, and red atoms for SF or HCP, depending on the thickness.

More specifically, the $J$ parameters of the representative pure metals^23^ and Cantor-Wu alloys were computed and shown in the 2D $J$ space. The $J$ space can be divided into three regions based on the energy of the structures described by the ANNNI model. Each region favors one of the FCC, HCP, and DHCP structures (Fig. 1b, c). For example, FCC alloys are located between ${J}_{1}=0$ and ${J}_{1}+2{J}_{2}=0$. Notably, the Cantor-Wu alloys distribute right around the line ${J}_{1}+2{J}_{2}=0$ (Fig. 1c). Fitting the data points to a linear equation, we obtain ${J}_{2}=-0.53{J}_{1}+1.66$ with Pearson’s ${R}^{2}=0.98$. The coefficient of −0.53 is close to −1/2, which indicates an intrinsic feature of these alloys. As will be shown later, this suggests that rich nanoscale stacking sequences of CPSs can exist in these alloys. One of its possible underlying physical origins will be elaborated in the next section and Fig. 2. The scale of ${J}_{1}$ axis in Fig. 1a is several times larger than ${J}_{2}$, demonstrating the dominance of ${J}_{1}$. In contrast to pure metals, the high alloying degree of the HEAs results in smaller interaction parameters. The possible origin is that the electrons per atom in this series of alloys make HCP and FCC energetically more similar. According to the ANNNI model, ${J}_{1}=({E}_{{HCP}}-{E}_{{FCC}})/2$, which approaches zero.

### The close-packed structures with various stackings

Albeit with only two interaction parameters, the ANNNI model describes enthalpies of arbitrary CPSs reasonably well^15,24–26^. Almost all applications of ANNNI models are for simple CPSs with short periods. Here we explore their potential for structures with more complex sequences by including extra variables accounting for periodicity that must be derived (see supplementary material). Figure 2a shows the schematic phase diagram for a three-dimensional ANNNI model in the ($\kappa,{J}_{1}$) space, which provides a useful theoretical tool for understanding the close-packed defects. We define $|\kappa |=|-{J}_{2}/{J}_{1}|$. When the sign of $\kappa$ is negative, stacking sequences comprised of FCC-like and HCP-like units are energetically preferred; when the sign is positive, stacking sequences comprised of HCP-like and DHCP-like units are energetically preferred. We focus on the general case when CPSs have close energies, regardless of which CPSs are preferred. The parameter is close to 1/2 for the multicomponent Cantor-Wu alloys with $|\kappa|\in$ [0.4,0.8]. The ANNNI model shows $|\kappa|$ =1/2 is unique in that even at zero temperature, modulated structures (i.e., CPSs different from FCC) are present due to the comparable strength of the nearest-neighbor and next-nearest-neighbor interactions. This insight is consistent with the low-temperature observation that rich deformation mechanisms with nanoscale activation length are active^16,17^. One of the fingerprint features of HEAs, i.e., the randomness in chemical distributions, results in a scattered $\kappa$, particularly for ultra-fine-grain materials with small plane areas (i.e., larger standard deviation *σ* in Eq. S1 of SFE). The scattered $\kappa$ allows some configurations to enter the DHCP region ($|\kappa|\, > \,0.5$), and others to enter the HCP ( ${J}_{1}\, < \,0$) or FCC ( ${J}_{1}\, > \,0$) regions. Therefore, the scattered $\kappa$, as a consequence of the randomness in configurations, is expected to promote the polymorphism of formed CPSs

We randomly sampled the CPSs using 24 close-packed layers as a model system with ${J}_{1}=$ −20 mJm^−2^, ${J}_{2}=$ 10 mJm^−2^, and $\kappa=$−0.5. This model system is like a typical Cantor-Wu alloy. The results are shown in Fig. 2b–d. After 3000 random steps, the energy landscape of this close-packed system is almost fully explored. This is seen more clearly in the (${n}_{1}$, ${n}_{2}$) space enclosed by the three lines between FCC, HCP, and DHCP in Fig. 2c, where ${n}_{1}=-{\sum }_{i}{S}_{i}{S}_{i+1}$ and ${n}_{2}=-{\sum }_{i}{S}_{i}{S}_{i+2}$. Most of the allowable configurations (represented by dots) are visited. Each CPS corresponds to one dot in Fig. 2c, such as one CPS shown in Fig. 2d. Figure 2a shows that different $\kappa$ promotes the formation of different stacking sequences of CPSs. Since $\kappa$ can be tuned chemically (i.e., by varying the element concentrations), this offers one pathway to control the nanoscale CPSs.

The outstanding combinations of strength and ductility in the multicomponent alloys are related to CPSs. Like twins and stacking faults (SFs)^16,18,27^, CPSs can also be formed through partial dislocations, multiple partial dislocations^16,18,28^. These structures can act as barriers to mobile dislocations and generally contribute to work hardening^27^. In experiments, abundant CPSs with various stackings are observed in deformed samples under high-resolution microscopies, such as nano twin and nano HCP layers in CoCrNi^6,16,18,29^. The nanoscale stacking sequences can impede dislocation gliding, thereby providing a strengthening effect. Also, forming these CPSs can introduce deformation-induced plasticity and enhance the ductility of HEAs. Interestingly, here we find the formations of CPSs also have thermodynamic origins, which is indicated by the linear distributions of the Cantor-Wu alloys in Fig. 1c.

### Criteria for HEA design and applications

The insights above suggest two criteria for designing HEAs with enhanced strength and ductility: (i) The ratio of interaction parameters $\left|\kappa \right|$ must be close to ½; and (ii) low to negative SFEs. Several examples meet these two criteria, such as VCoNi^30^ and the Cantor-Wu alloys^6^. Both requirements can be realized by tuning the alloy composition and thus can be employed to design alloys with excellent strength and ductility. Following these guidelines, six Co-Cr-Fe-Ni-Mo HEAs are proposed (i.e., alloys A35, A36, A61, A62, A5, and A6, see Table 1, Figs. S3, and 3). As a natural choice, we start with Mo and four principal elements of the Cantor-Wu alloys by mainly changing the concentration of Ni and Mo. Ni promotes FCC-like stacking, and tunning the Ni concentration can change both SFE and the kappa parameter. The choice of adding Mo resides in its potential to change the $\kappa$ parameter and SFEs via lattice distortion while at the same time enhancing the pitting corrosion resistance. The alloys A36, A62, and A6 contain Mo, and the other three are designed for comparison.

**Table 1:** The DFT-computed stacking fault energies (SFEs) for different configurations

Alloy IDs	A35	A36	A61	A62	A5	A6
Alloy	CoCr FeNi_2_	CoCrFeNi_2_Mo_0.25_	CoCr_1.25_FeNi_3_	CoCr_1.25_FeNi_3_Mo_0.5_	CoCr_1.5_FeNi_4_	CoCr_1.5_FeNi_4_Mo_0.5_
SFE	−7.8	−12.7	−19.7	−52.9	12.2	−52.0
${{{{{\boldsymbol{|}}}}}}{{{{{\boldsymbol{\kappa }}}}}}{{{{{\boldsymbol{|}}}}}}$	0.03	0.42	0.00	0.33	0.18	0.26

The SFEs of the spin-polarized calculations are summarized here for the six HEAs. All SFEs are in mJm^−2^. Also shown are the ratios of the interaction parameters *κ*.

**Fig. 3:**
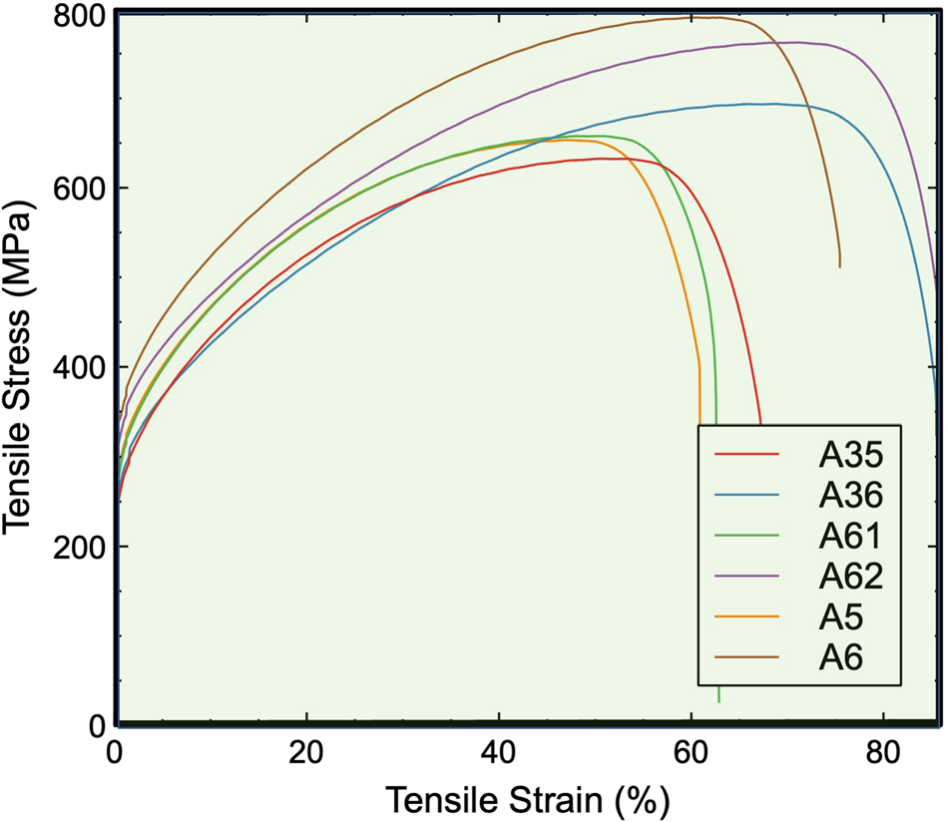
The mechanical behavior of the six designed HEAs at room temperature. The alloys A35, A61, and A5 without Mo are intentionally designed in comparison to the corresponding A36, A62, and A6 alloys with Mo. Apparent differences are seen in the ultimate tensile strength, ductility (strain to failure), and toughness (area under the curve) between the two groups of alloys. The alloys were synthesized following similar procedures (see “Methods” section part) and had comparable grain sizes of 20 µm (Fig. S1). This grain size is close to those of the Cantor-Wu alloys, between 24 and 48 µm^6^. The mechanical properties with error bars are tabulated Table S2 in the supplementary material, and the 18 tensile-test curves are shown in Fig. S6.

**Fig. 4:**
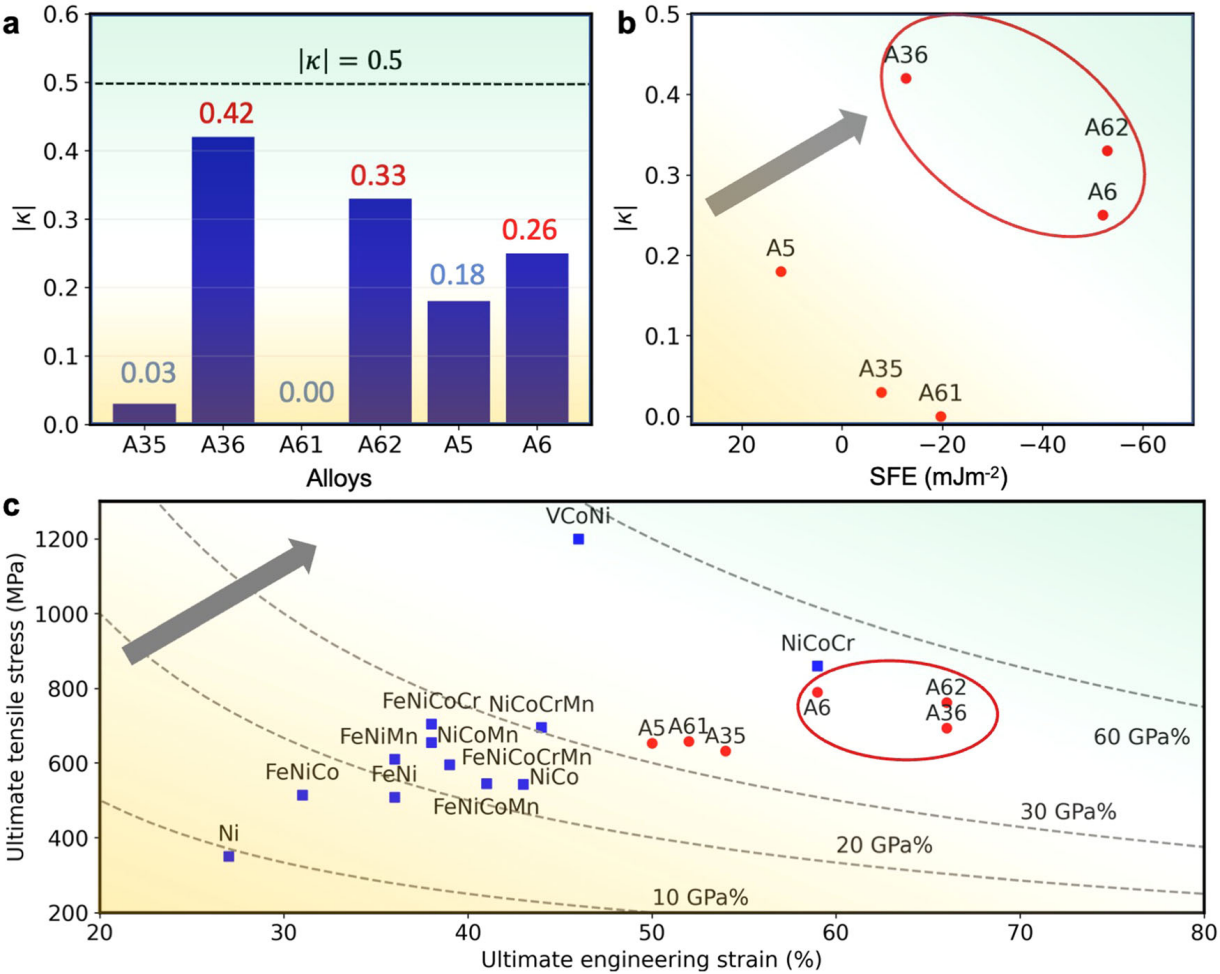
The stacking fault energies (SFEs) and associated physical quantities for the 6 HEAs. **a** The $\kappa$ parameter calculated by DFT; **b** The ratios $|\kappa|$ of the interaction parameters for the 6 HEAs. There are alloys with $|\kappa|$ ratios closer to ½ than the others, i.e., A36, A62, and A6. **c** The ultimate tensile stresses and ultimate strains at room temperature for the six HEAs and the Cantor-Wu alloys, with alloys A6, A62, and A36 circled and highlighted. The ductility-strength tradeoff is obtained for alloys with negative SFEs and |$\kappa|$ close to ½. The dashed lines are used to guide the eyes and have the same meaning as in Fig. 1a. The gradient color in the background indicates the change of the physical quantities (e.g., the SFE, ultimate tensile stress, etc.) corresponding to each axis.

The temperature-dependent phase fractions calculated by CALculations of PHAse Diagram (CALPHAD) method are shown in Fig. S3 for these alloys. All alloys possess a wide temperature range for single-phase FCC, albeit with slightly different starting temperatures, indicating they are promising candidates for FCC solid solutions. Table 1 shows their constitutions, as well as their SFEs and $\kappa$. These alloys are manufactured, deformed, and characterized following the procedure described in the Method part. The SFEs of these HEAs with and without Mo are calculated using DFT methods in both spin-polarized and non-polarized cases (see supplementary material). Since the spin-polarized states are more stable than the nonmagnetic (NM) ones for these HEAs and the trend is similar for both magnetic states, only the spin-polarized results are adopted for further discussion herein. Except for alloy A5, whose SFE is a small positive number, the other five alloys all have negative SFEs. Molybdenum significantly reduces the already negative SFEs (see Table 1 and Fig. 4(b)). This trend holds for all three pairs of HEAs, i.e., (A35, A36), (A61, A62), and (A5, A6). While the SFE increases slightly upon Mo addition, more Ni generally increases SFEs, a trend among the HEAs without Mo (A35, A61, and A5). These results show that criterion (i) can be realized without difficulty.

The ratios $\kappa$ for these alloys are shown in Fig. 4a. Since we use a finite supercell to model solid solutions, a reliable *κ* parameter must be calculated by sampling sufficient configurations when its value converges. The associated convergence tests are conducted in Fig. S4. Since the $|\kappa|$ is calculated by first averaging and then taking the absolute value of the average, both $\kappa$ and $|\kappa|$ converge at the same time. The alloys with $|\kappa|$ close to ½ are A62, A6, and A35. The SFEs of five alloys are negative, and only Alloy A5 with a positive SFE. These alloys are arranged in the two-dimensional SFE- $\kappa$ space in Fig. 4b. The alloys A36, A62, and A6 appear in the upper right corner of the figure. They meet criterion (ii) better than others, with $|\kappa|\in [{{{{\mathrm{0.26,0.42}}}}}]$. Furthermore, their SFEs are also very negative. As stated above, alloys that meet criteria (i) and (ii) have an energetic tendency towards forming nanoscale stacking domains distinct from FCC, which we propose is beneficial to increasing work hardening. In addition, the two criteria suggest an increase of ductility by activating the generalized deformation-induced plasticity mechanism, which differs from but is like TWIP and TRIP. However, more experimental work is needed to validate this theoretical argument, a topic for our future work. Therefore, they are expected to have the best ultimate strength and ductility in experimental tests. The alloys A35, A61, and A5 meet either only one or neither of the two criteria. Therefore, they may have mechanical properties inferior to the other three alloys.

Figure 4c and Fig. S6 summarize the results of the mechanical behavior of these HEAs. Full tensile test curves have been shown in Fig. 3. The ultimate tensile strengths and strains shown in Fig. 4c indeed validate our theoretical predictions and are highly correlated with the locations of the same alloys in Fig. 4b. The best combination of ultimate tensile strengths with ductility is obtained in alloys A36, A62, and A6. With comparable grain sizes (i.e., 20 µm, see Fig. S1), the three alloys exhibit similar or even better strength-ductility tradeoffs than CoCrNi, the best alloy among the Cantor-Wu series. The A61 alloy has a very negative SFE, but its $|\kappa|$ ratio is almost zero; the $|\kappa|$ ratio of A5 is not very small, but its SFE is a positive number. In A35, both criteria are not well satisfied, i.e., its SFE is only a slightly negative number, and the $|\kappa|$ ratio is close to zero, i.e., far below ½.

## Discussion

The CPSs promote the activation of a TWIP-like mechanism. To differentiate it from the TWIP, we coin a term, the generalized TWIP effect. The generalized TWIP mechanism introduces freedoms to accommodate plastic deformation and overcome the strength-ductility tradeoff^4,18^. The connection between the parameter $|\kappa|$ and the work hardening can be demonstrated using the microscale dislocation theory. In alloys with abundant defects introduced during either sample preparation or plastic deformation, the critical shear stress of a dislocation $\mu$ at a specific temperature is^31,32^

1 $${\tau }_{c}^{\mu }=G{b}^{\mu }\sqrt{{\sum }_{\nu }{A}^{\mu \nu }{\rho }_{f}^{\nu }},$$

where ${\rho }_{f}^{\nu }$ represents the density of dislocations, SFs, twins, nanoscale HCP, or other CPSs $\nu$ that can act as obstacles (like “forest” dislocations) for the mobile dislocation $\mu$. ${b}^{\mu }$ is the Burgers vector of the dislocation $\mu$, $G$ is the shear modulus, and ${A}^{\mu \nu }$ is the interaction parameter between $\mu$ and $\nu$. A higher density of nanoscale stacking sequences results in a larger ${\rho }_{f}^{\nu }$, as well as higher critical shear stress ${\tau }_{c}^{\mu }$ for mobile dislocations. Theoretically, the parameter $\kappa$ that can control the defect density $\rho$ introduces an independent criterion for HEA design on top of SFEs.

Taking alloys A6 and A35 as examples, we study the deformation mechanisms and characterize the microstructure of newly designed HEAs guided by the *κ* parameter. These experimental details can further assess our theory proposed at the micro- to the nanoscale. The electron backscattered diffraction (EBSD) and twin boundary mappings (Fig. S2a, b) show a high density of twins and twin-like nanoscale CPSs in A6 (Fig. S2a), similar to CoCrNi^16^. In contrast, these defects are significantly fewer in density in A35 (Fig. S2b). When $\left|\kappa \right|$ approaches ½, i.e., for the A6 alloy, a high density of ${\rho }_{f}^{\nu }$ is expected (e.g., Fig. S2a for twin boundaries), which contributes significantly to the overall high work hardening (${\tau }_{c}^{\mu }$). The HRTEM images of A6 are consistent with our prediction, containing a high density of defects, as seen in Fig. S2c. The fractured A6 sample has a high density of defects produced by deformation-induced plasticity. The defects are usually partial dislocations/stacking faults consistent with the ultralow SFEs and $\left|\kappa \right|\sim$½. The stacking defects in the form of SFs, twins, and other stacking anomalies are observed on the {111} planes in Fig. S2d–f. These defects act as barriers to impede mobile dislocations and increase the strength of the alloys. Furthermore, they can interact to form Lomer-Cottrell locks, which are observed here. Nonetheless, Fig. S2 cannot be directly compared with our computational prediction in Fig. 2. The motivation for showing Fig. 2 is mainly to explain our alloy-design principle based on the $\kappa$ parameter. One should note that the prediction in Fig. 2 is based on the assumption of a thermodynamic equilibrium condition at zero Kelvin, which is different from the casting condition of our alloys; therefore, the precise experimental verification of the simulation is very challenging.

We only consider the zero-temperature $\kappa$ parameter that allows for efficient screening and designing of HEAs. There are two reasons for this choice. First, including temperature effects requires expensive calculations of many entropic terms^10,16^. In addition, previous finite-temperature studies usually focused on one alloy rather than many alloys in a high-throughput manner, e.g., CoCrFeNiMn^10^ and CoCrNi^16^. Second, it requires an accuracy of the order of meV/atom that is very challenging to achieve, limiting the benefit of including temperature^16,33^. Therefore, including temperature effects in the model is computationally impractical for the motivation here of an efficient screening. Instead, we use experimental measurements to supplement our model and validate the effectiveness of the $\kappa$ parameter with the designed HEAs synthesized in the experiment (Figs. 3 and 4). Nonetheless, it is worth mentioning that the energy volatility depends on the supercell size used to sample HEAs. Our method needs a sufficient number of randomly generated configurations to reliably represent the HEAs, for which convergence tests are needed.

In summary, we identify a fact that the multicomponent Cantor-Wu alloys with excellent mechanical properties (i.e., high strength and good ductility) have a ratio $|\kappa|$ of interaction parameters close to ½, a number that promotes the formulation of nanoscale stacking sequences in CPSs. The CPSs promote a generalized TWIP effect to overcome the strength-ductility tradeoff and enhance the toughness of HEAs. This parameter provides a more accurate quantifiable method jointly with the conventional SFE parameter to predict toughness. It offers a practical method to design high-toughness HEAs. Exemplary alloys were designed, manufactured, and characterized, which validated our theory and discovered alloys with enhanced toughness and better ductility than CoCrNi. The guiding parameter and the mechanism proposed here pave the pathway toward accurate prediction of combined strength and ductility in high-entropy alloys.

## Methods

### Sample preparation

The alloys were manufactured using a traditional cast and wrought processing route using high-purity industry grade stock material as well as master alloys. The melt charges were loaded in an alumina crucible for vacuum induction melting (VIM) under 200 Torr Ar partial pressure. The liquid was heated to a 50 °C superheat temperature before pouring into a 75 mm diameter graphite mold to obtain a ~ 8 kg cylindrical ingots. All ingots were homogenized in a vacuum heat treatment furnace under 50 Torr Ar partial pressure and utilizing Ar forced gas fan cooling. Following homogenization, the ingot surfaces were finished on a lathe for hot working. Predetermined steps of forging followed by hot rolling were used with reheat times between each pass to produce 10 mm thick plates of equiaxed grain structure. The last reheat was used as a solution heat treatment.

### Mechanical test

Mechanical test specimens were cut from the plates and consisted of reduced gage cylindrical samples of 76 mm in overall length and 10 mm in overall diameter with ANSI 3/8 × 16 threaded ends. The reduced gage section measured 32 mm in length and 6.3 mm in diameter. Tension testing was performed at various temperatures according to the ASTM E-8 standard.

### Microstructural characterization

The deformation microstructure of the alloys is characterized by electron backscattered diffraction (EBSD), transmission electron microscopy (TEM), and scanning transmission electron microscopy (STEM). Microscopy was performed at the National Center for Electron Microscopy, Molecular Foundry, Lawrence Berkeley National Laboratory. EBSD experiments were conducted using a ThermoFisher’s Strata SEM equipped with an EDEX EBSD detector. The deformed samples were sectioned from the as-fractured ASTM tensile specimen, which were then polished to a surface roughness of ~50 nm by standard metallographic methods. Conventional TEM and high-resolution TEM were performed using a ThermoFisher’s Titan X microscope operated at 300 kV. High angle annular dark field, atomic resolution STEM imaging was performed using the aberration-corrected TEAM I microscope operated at 300 kV with a convergent semi-angle of 30 mrad and camera length of 115 mm. The TEM/STEM samples were prepared using a ThermoFisher Helios G4 dual-beam focused ion beam facility. 30 kV focused Ga beam was used for the initial thinning and 2 kV for the final polishing.

For grain size measurement, please see supplementary material.

### DFT calculations

Spin-polarized and non-spin-polarized density functional theory (DFT)^34,35^ simulations were carried out using Vienna Ab-initio Simulation Package (VASP)^36^ to obtain the total energies for the SFE calculations. The generalized gradient approximation (GGA) parametrized by Perdew-Burke-Ernzerhof (PBE)^37^ was used to calculate the electronic exchange-correlation interaction and the Kohn-Sham equation was solved using the projector augmented wave (PAW) method^38^, with the Brillouin zone sampled using Monkhorst-Pack scheme^39^. The atomic configurations of elements in the pseudopotentials used in all calculations were Co [Ar]3d^8^4s^1^, Cr [Ar]3d^5^4s^1^, Fe [Ar]3d^7^4s^1^, Ni [Ar]3d^8^4s^2^, and Mo [Kr]5s^1^4d^5^. The relaxation stops when the energy difference between ionic steps is smaller than 10^−4^ eV. The SFEs are calculated using a supercell size of 72 atoms [4(1/2[110])×3(1/2[1-12]) × 6(1/3[1-1-1])] with the periodic boundary condition. The supercell is fixed at its optimal volume and shape for each alloy, and atoms are freely relaxed without restrictions. In spin-polarized calculations, the initial magnetic moments are aligned in the same direction (the third direction of the supercell). Atoms are randomly distributed in lattice sites for one sample (i.e., configuration). We consider more than 20 configurations to consider the randomness fully. A plane wave cutoff of 350 eV and the k-point meshes of 6$\times$ 4$\times$4 for the Brillouin zone were used. With an increase of k-point meshes by eight times (2$\times$2$\times$2), the change in total energy was less than 2 meV or 0.028 meV/atom.

### ANNNI model

The energies of the three basic structures given by the ANNNI model are ${E}_{{FCC}}={J}_{0}-{J}_{1}-{J}_{2}+O({J}_{2})$, ${E}_{{HCP}}={J}_{0}+{J}_{1}-{J}_{2}+O({J}_{2})$, ${E}_{{DHCP}}={J}_{0}+{J}_{2}+O({J}_{2})$. The interaction parameters ${J}_{i}$ can be determined from the DFT-computed energies of FCC, HCP and DHCP systems by solving these three equations.

For profuse close-packed structures like SFs or twins, their energies can be written similarly. Subtracting the energy of the matrix from the defect energy and dividing it by the faulted plane area, the faulted energy in J/m^2^ can be obtained. Differently, for close-packed structures of arbitrary periods, extra parameters *n* was introduced to denote the number of periods for the repeated units. Details are included in the supplementary material.

### Random sampling

We propose a random sampling based on the ANNNI model to mimic the possible close-packed structures at $\left|\kappa \right |=0.5$. A model system of 24 close-packed layers (see Fig. 2) was chosen. The relation between every two adjacent layers was determined by a randomly generated number −1 or 1. The last number determines its relationship with the first layers, so the periodic boundary condition was adopted. The 24 random numbers determine the system energy through the ANNNI model and the local features of the close-packed stacking. For example, the normal FCC stacking is in green, twin layers in blue, and HCP and stacking faults are in red. HCP layers and stacking faults are differentiated by the number of layers. After about 3000 random steps, the allowable geometric and energy space are almost fully explored.

### Other technical details

The derivation of the model to connect SFE and misfit volumes is a mathematically tedious procedure. Details of this process are included in the supplementary material. Also, the evaluation of the error bars for SFEs is demonstrated in the supplementary material.

## Supplementary information

Supplementary Information

## Data Availability

The data used in this study are provided in the Supplementary Information. All other data related to the manuscript are available from the corresponding authors upon request.
