# Supplementary material for: Theory-guided design of high-entropy alloys with enhanced strength-ductility synergy
Source: Nat Commun. 2023 May 2;14:2519. doi: 10.1038/s41467-023-38111-6 (PMC10154317; doi:10.1038/s41467-023-38111-6)
Supplement: Supplementary file 1 — Supplementary Information [file 41467_2023_38111_MOESM1_ESM.pdf]

Supplementary Methods
Theory-guided design of high-entropy alloys with enhanced strength-ductility synergy

Zongrui Pei, Shiteng Zhao, Martin Detrois, Paul D. Jablonski, Jeffrey A. Hawk, David E. Alman, Mark Asta, Andrew M. Minor, Michael C. Gao

April 8, 2023

1 Experiment of Characterization

1.1 Materials Characterization

We measure the grain sizes of the designed alloys, which were synthesized following a similar procedure (see Methods part) and have comparable grain sizes. As representative examples, grain-size distributions of the A6 and A35 are shown in Figure 1. Most of the grains have a size of around 20 micrometers.

1.2 Characterization of microstructure

The deformation microstructure of the alloys is characterized by electron backscattered diffraction (EBSD), transmission electron microscopy (TEM), and scanning transmission electron microscopy (STEM). The images are summarized in Figure 2.

2 Modeling and Simulations

2.1 CALPHAD

CALculations of PHAse Diagram (CALPHAD) calculations are carried out using TCNI8 thermodynamic database provided by ThermoCalc^{TM} [1]. The temperature-dependent phase fractions are shown in Figure 3 for the six high-entropy alloys. All alloys show a wide temperature range for single-phase FCC albeit with different starting temperatures.

2.2 The error bar of SFEs

DFT-computed SFEs and J parameters usually have substantial fluctuations due to the finite-size supercells. For a typical FCC alloy with the nearest-neighbor distance $a = 2.5$Å, a supercell of $3 \times 3 \times 8$ (72 atoms) corresponds to a SF area of $S_{th} \approx 9 \times \frac{\sqrt{3}}{2}a^2 \approx 0.5\text{nm}^2$

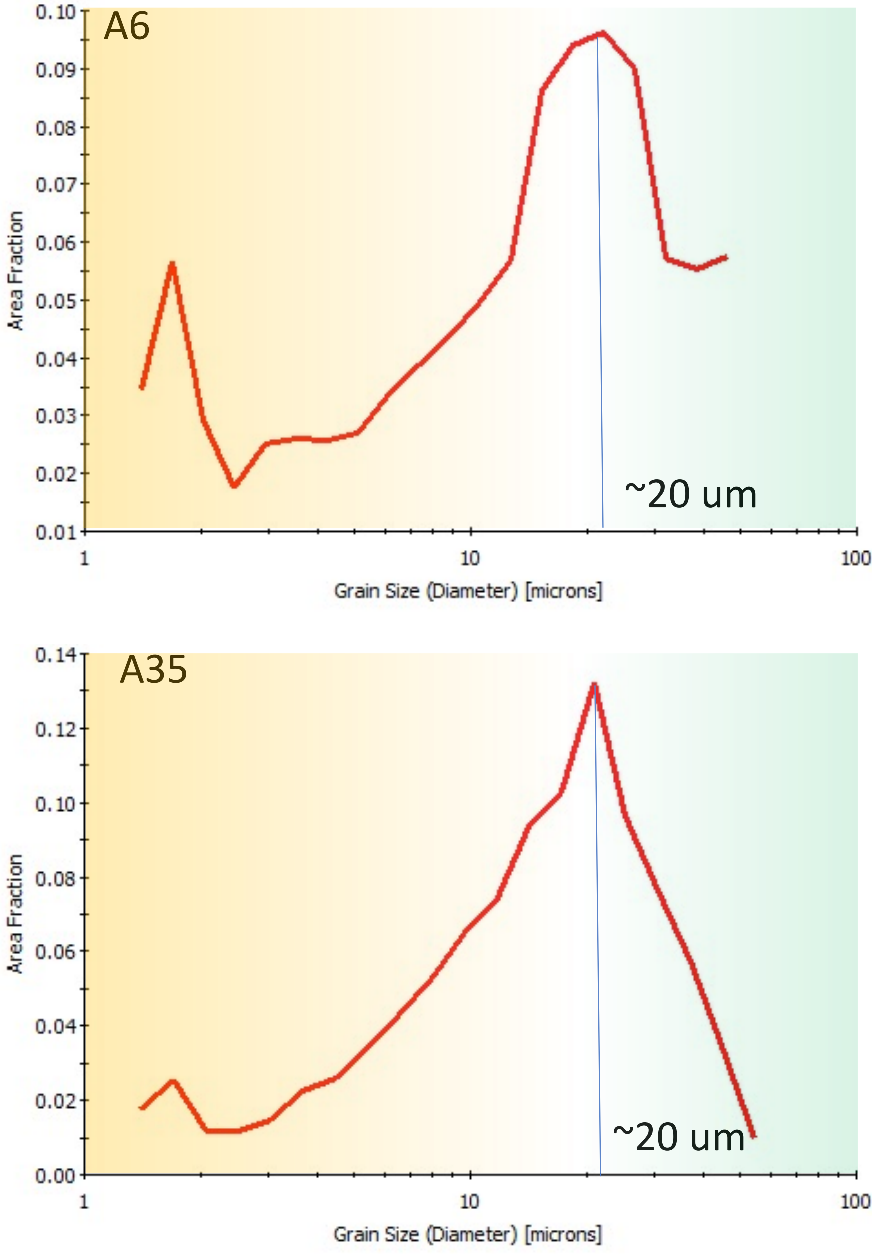

Supplementary Figure 1: Grain size measurements of two representative high-entropy alloys.

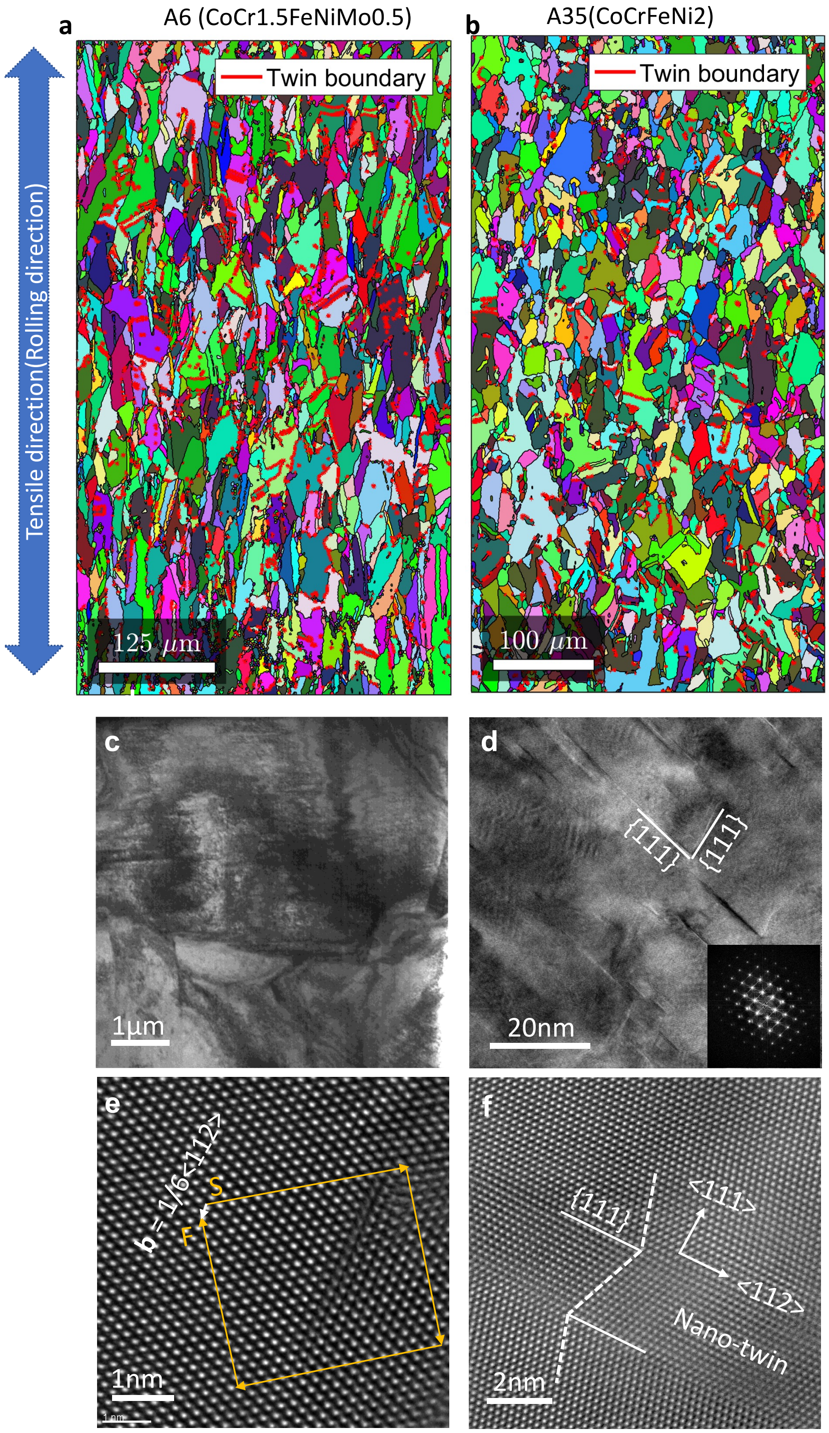

Supplementary Figure 2: Experimental characterization of the HEAs after tensile loading. a-b, Electron backscatter diffraction (EBSD) images of A6 (a) show a much higher density of twin boundaries than A35 (b). c, Low magnification TEM micrograph of the as-deformed A6 sample, showing high density of defects; d, Intermediate magnification TEM micrograph of A6 sample showing that defects are primarily located on the {111} planes; e, High-resolution TEM micrograph of A6 showing one stacking fault with a partial dislocation; f, The high-angle-angular-dark-field (HAADF) image of a nanoscale twin.

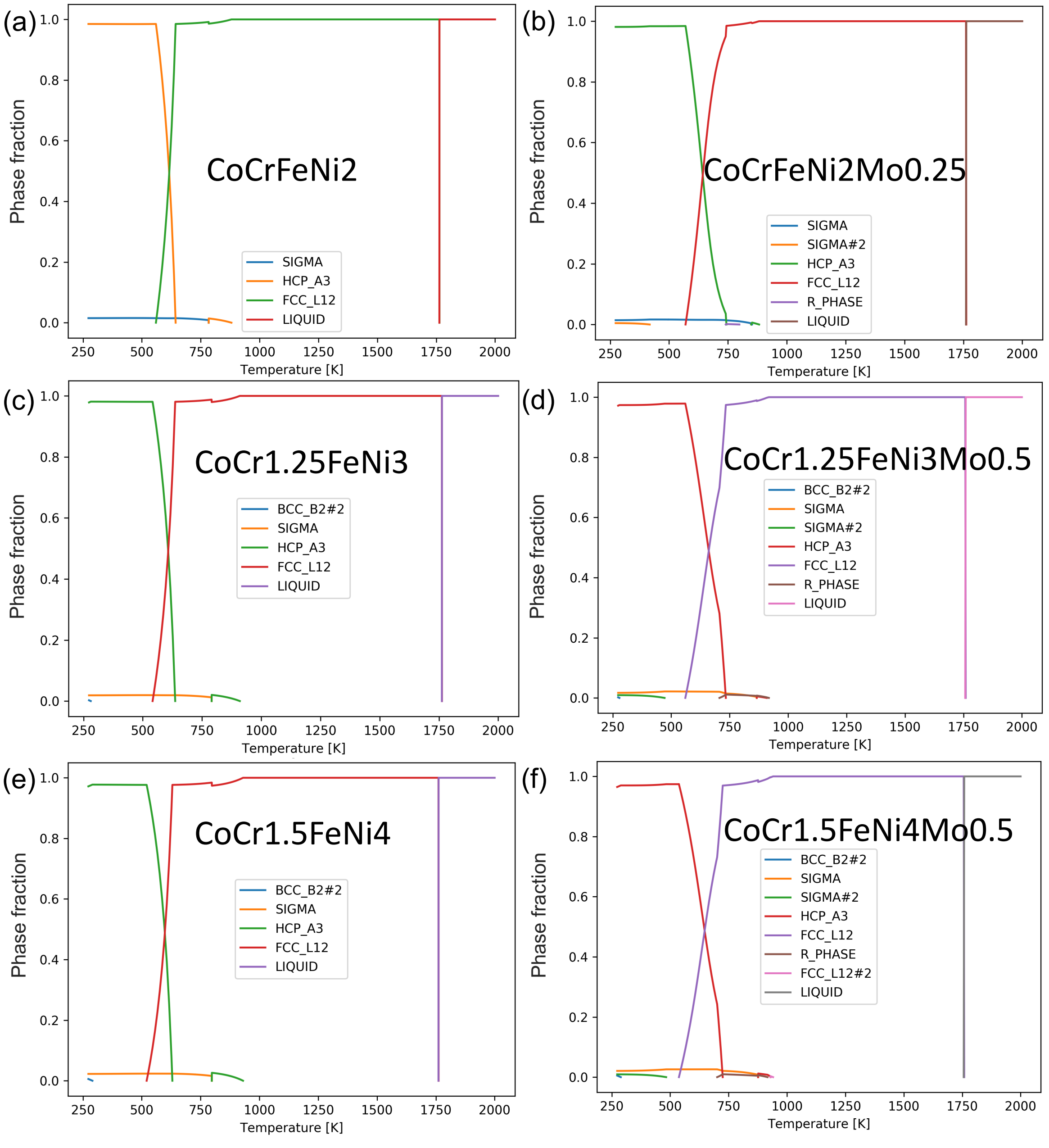

Supplementary Figure 3: CALPHAD calculations for six high-entropy alloys with and without Mo. There exist wide temperature ranges for single FCC phases for all the alloys.

and the standard deviation of $\sigma_{th} \leq 70$ mJ/m^2. However, in experiment the grain size D is of the order of 1μm [2, 3], so $S_{exp} \approx D^2 \approx 1\mu$m^2. Theoretically, the standard deviation σ is inversely proportional to the square root of SF area [4, 5]. The actual fluctuation for SFEs in common grain sizes of 1μm is

$$\sigma_{exp} = \sigma_{th}\sqrt{\frac{S_{th}}{S_{exp}}} \approx 0.1\text{mJ/m}^2, \tag{1}$$

which is 2-3 orders of magnitude smaller than SFEs. The J parameters are linear functions of SFEs in ANNNI model. It is reasonable to assume the J parameters have the same small standard deviations. In the figures involved J parameters, the error bars are not shown.

2.3 The convergence of $|\kappa|$

As discussed in the last section, the standard deviations of DFT results for SFEs or $|\kappa|$ are not error bars, but statistical properties of the finite supercell model [4] when the sufficient number of calculations are considered. The standard deviations will not become increasingly smaller but a constant for a supercell. It does become smaller when a larger supercell is used. Unlike the standard deviation, the expectation is not sensitive to supercell size. It becomes convergent when a sufficient number of calculations are performed. We take the $|\kappa|$ of A5 as an example to demonstrate this argument in Figure 4.

2.4 The generalized ANNNI models

The energies of the three basic structures given by ANNNI model are

$$E_{FCC} = J_0 - J_1 - J_2 + O(J_2), \tag{2}$$
$$E_{HCP} = J_0 + J_1 - J_2 + O(J_2), \tag{3}$$
$$E_{DHCP} = J_0 + J_2 + O(J_2). \tag{4}$$

The energies can be written similarly for profuse close-packed structures like SFs or twins. By subtracting the energy of the matrix from the defect energy and dividing it by the faulted plane area, we can obtain the faulted energy in J/m^2. Differently, we have to introduce extra parameters to denote the number of periods for the repeated units for close-packed structures of arbitrary periods. For simplicity, we consider the simplest case with only one repetitious unit of varying periods.

We denote the thickness of HCP stacking by n in the following two representative cases:

$$(i)...ABCABC(AB)^nABCABC... \tag{5}$$
$$(ii)...ABCABC(AB)^nCBACBA... \tag{6}$$

The first case is for n-period nano-HCP embedded in the FCC matrix, while the second is for the same repetitious unit embedded by a FCC twin. We derive the following equations for the two cases within the Next-Nearest-Neighbor (NNN) approximation:
(i) Nano-scale HCP located at FCC twin boundary,

$$\Delta F_i(n,T) = F_i(n,T) - F_{FCC} = (4n+2)J_1(T) + 4J_2(T); \tag{7}$$

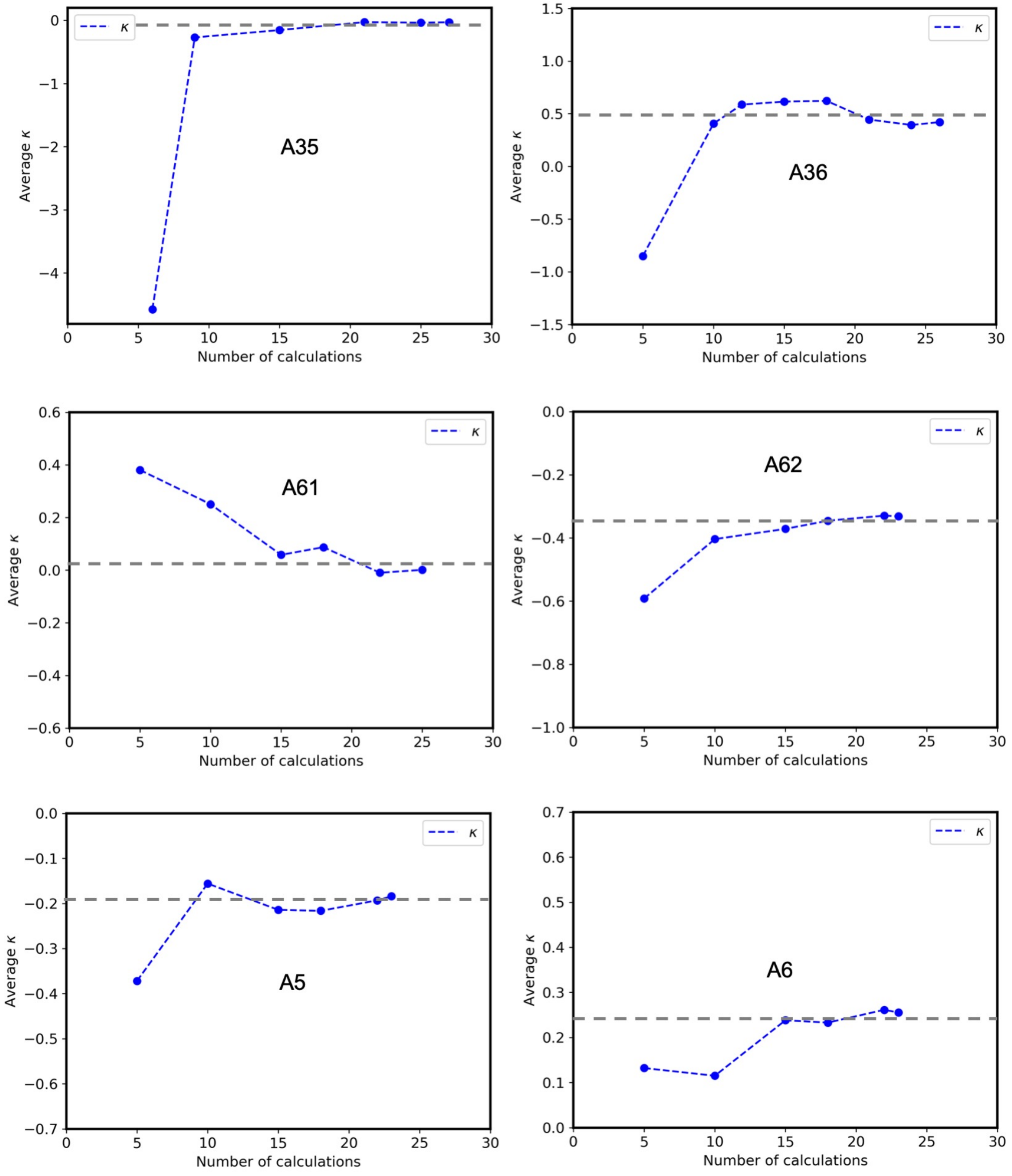

Supplementary Figure 4: The convergence tests of κ ($|\kappa|$) for the six new alloys. The κ becomes a constant with an increasing number of calculations. For example, a reliable κ can be obtained with 15 calculations. It becomes a constant above 15 calculations.

(ii) Nano-scale HCP in FCC matrix,

$$\Delta F_{ii}(n,T) = F_{ii}(n,T) - F_{FCC} = 4nJ_1(T) + 4J_2(T). \quad (8)$$

Here we use F instread of E, since we write the energy and interaction parameters as functions of temperature. They can be rewritten using the expressions of HCP, FCC and DHCP:

(i) Nano-scale HCP layers located at FCC twin boundary,

$$\Delta F_i(n,T) = 2(E_{DHCP} - E_{FCC}) + 2n(E_{HCP} - E_{FCC}) \quad (9)$$

(ii) Nano-scale HCP layers in FCC matrix,

$$\Delta F_{ii}(n,T) = 2(E_{DHCP} - E_{FCC}) + (2n-1)(E_{HCP} - E_{FCC}) \quad (10)$$

2.5 Tabulated data

All the data plotted in Figure 1 of the main text are tabulated in the table of this section.

3 Tensile tests

Tensile tests for the six high-entropy alloys were performed three times for each of the alloys. The complete tensile tests are shown in Figure 5. Key data points were extracted and tabulated in Table 2.

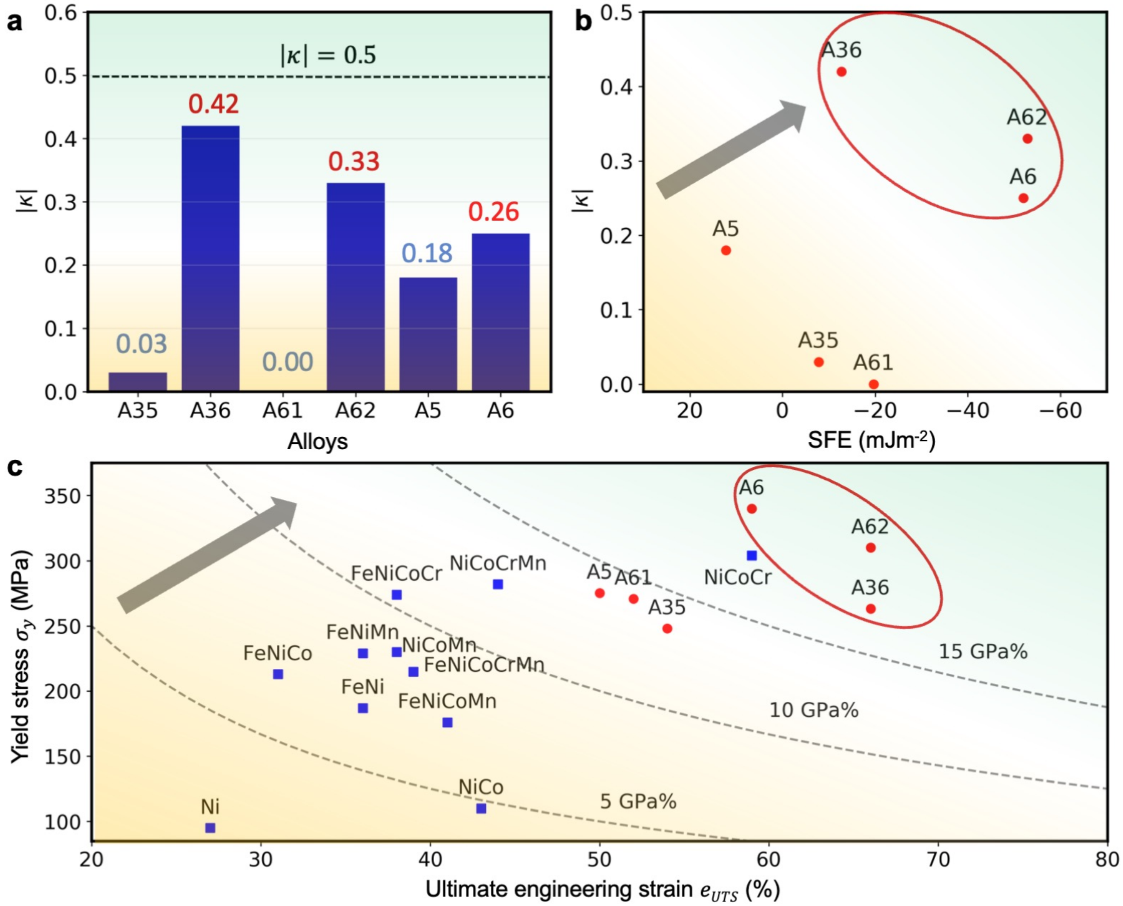

Supplementary Figure 5: The stacking fault energies (SFEs) and associated physical quantities for the 6 HEAs. a, SFEs computed in ferromagnetic (FM) states; b, The ratios $|\kappa|$ of the interaction parameters for the 6 HEAs. There are alloys with $|\kappa|$ ratios closer to 0.5 than the others, i.e., A36, A62, and A6. c, The yield stresses and ultimate strains at room temperature for the six HEAs along with the Cantor-Wu alloys, with alloys A6, A62 and A36 circled and highlighted. Negative SFE puts six alloys in a good position in a strength-ductility tradeoff, but κ differentiates them further.

Supplementary Table 1: The experimental mechanical properties and theoretical SFEs collected from both literature and our present calculations. Solid solution strengthening σ_{ss} in MPa, ultimate tensile strain (UTS) in %, grain size D in μm, locking parameters k in MPa$\sqrt{\mu m}$. For consistency, the SFEs of the Cantor-Wu alloys are taken from Ref. [6], with validation of our own DFT calculations. The SFEs of the rest alloys are calculated in this study.

Alloys	σ_{ss}	UTS/%	SFE	grain size D	yield stress	k	Ref.
FeCoCrNiMn	125.4	39.0	-52.0	-	215.0	-	Wu2014 [2]
FeCoCrNi	97.8	38.0	-21.6	-	274.0	-	Wu2014 [2]
FeNiCo	85.2	31.0	56.5	-	213.0	-	Wu2014 [2]
CoCrNi	130.4	59.0	-61.2	-	304.0	-	Wu2014 [2]
FeNi	80.8	36.0	105.6	-	187.0	-	Wu2014 [2]
NiCo	5.8	43.0	-10.2	-	110.0	-	Wu2014 [2]
FeCoCrNiMn	125.0	60.0	-52.0	4.4	167.1	494.0	Otto2013 [7]
FeCoCrNiMn	125.0	78.0	-52.0	155.0	355.7	494.0	Otto2013 [7]
FeCoCrNi	138.0	49.0	-21.6	1 2	540.0	492.1	Zaddach2015 [8]
FeCoCrNi	138.0	82.0	-21.6	60 80	197.0	492.1	Zaddach2015 [8]
Ni37.5Cr25Co37.5	184.6	62.2	-2.0	60.0	244.4	250.0	Coury2018 [9]
Ni37.5Cr25Co37.5	184.6	-	-2.0	20.0	290.0	250.0	Coury2018 [9]
CoCrNi	257.8	55.5	-61.2	-	356.5	253.0	Coury2018 [9]
Ni27.5Co27.5Cr45	404.7	49.0	-39.0	80.0	560.5	489.0	Coury2018 [9]
VCoNi	379.0	55.0	-34.7	27.8	520.8	864.0	Sohn2019 [10]
VCoNi	379.0	46.0	-34.7	5.6	766.6	864.0	Sohn2019 [10]
VCoNi	379.0	38.0	-34.7	2.0	991.6	864.0	Sohn2019 [10]
Fe80-xMnxCo10Cr10	202.0	74.0	-24.0	4.5	338	288.5	Li2016 [11]
Fe80-xMnxCo10Cr10	202.0	50.0	-24.0	45	245	288.5	Li2016 [11]

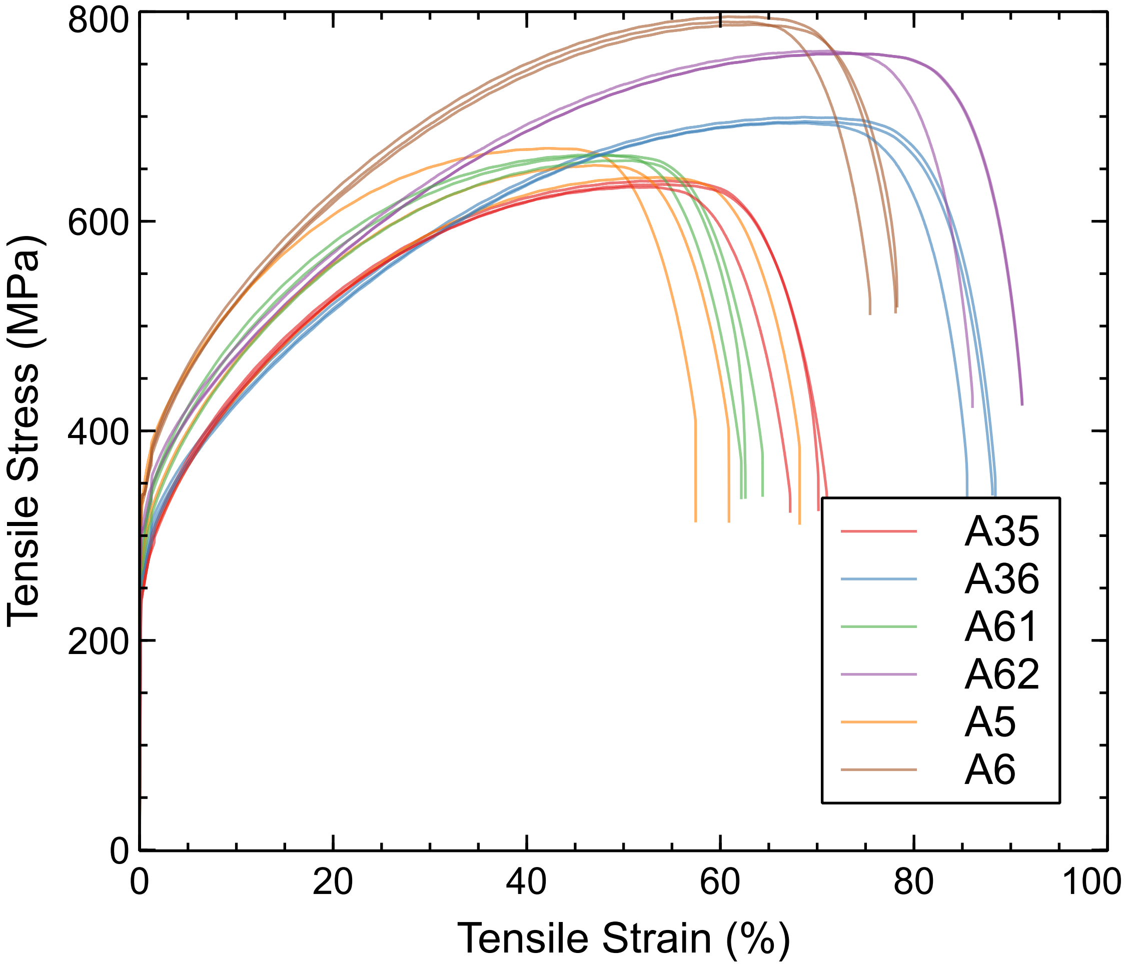

Supplementary Figure 6: The mechanical behavior of the six designed HEAs at room temperature.

Supplementary Table 2: The tensile tests of the six high-entropy alloys.

Alloy	Test	UTS (MPa)	0.2% YS(MPa)	Elongation to Failure (%)
A35	#1	632	248	54
	#2	635	247	58
	#3	638	251	58
	Ave ± Stdev	635 ± 3	249 ± 2	57 ± 2
A36	#1	694	263	66
	#2	695	261	69
	#3	699	266	68
	Ave ± Stdev	696 ± 3	263 ± 2	68 ± 2
A61	#1	658	271	52
	#2	663	289	53
	#3	663	281	53
	Ave ± Stdev	661 ± 3	281 ± 9	53 ± 1
A62	#1	762	310	66
	#2	760	306	71
	#3	760	304	70
	Ave ± Stdev	761 ± 1	307 ± 3	69 ± 2
A5	#1	653	275	50
	#2	642	249	54
	#3	670	335	49
	Ave ± Stdev	655 ± 14	286 ± 44	51 ± 3
A6	#1	790	340	59
	#2	795	340	62
	#3	787	332	61
	Ave ± Stdev	791 ± 4	337 ± 5	60 ± 1

Supplementary References

[1] Sundman, B., Jansson, B. & Andersson, J.-O. The thermo-calc databank system. *Calphad* **9**, 153–190 (1985).

[2] Wu, Z., Bei, H., Pharr, G. & George, E. Temperature dependence of the mechanical properties of equiatomic solid solution alloys with face-centered cubic crystal structures. *Acta Materialia* **81**, 428 – 441 (2014). URL http://www.sciencedirect.com/science/article/pii/S1359645414006272.

[3] Yu, P. *et al.* Phase transformation assisted twinning in a face-centered-cubic fecrnicoal0.36 high entropy alloy. *Acta Materialia* **181**, 491 – 500 (2019). URL http://www.sciencedirect.com/science/article/pii/S135964541930672X.

[4] Pei, Z. Theory of the energy fluctuation of multicomponent alloys. *Scripta Materialia* **162**, 503 – 506 (2019). URL http://www.sciencedirect.com/science/article/pii/S1359646218307371.

[5] Zhao, S., Osetsky, Y., Stocks, G. M. & Zhang, Y. Local-environment dependence of stacking fault energies in concentrated solid-solution alloys. *npj Computational Materials* **5**, 13 (2019). URL https://doi.org/10.1038/s41524-019-0150-y.

[6] Zhao, S., Stocks, G. M. & Zhang, Y. Stacking fault energies of face-centered cubic concentrated solid solution alloys. *Acta Materialia* **134**, 334 – 345 (2017). URL http://www.sciencedirect.com/science/article/pii/S1359645417303671.

[7] Otto, F. *et al.* The influences of temperature and microstructure on the tensile properties of a cocrfemnni high-entropy alloy. *Acta Materialia* **61**, 5743–5755 (2013).

[8] Zaddach, A., Scattergood, R. & Koch, C. Tensile properties of low-stacking fault energy high-entropy alloys. *Materials Science and Engineering: A* **636**, 373–378 (2015).

[9] Coury, F. G., Clarke, K. D., Kiminami, C. S., Kaufman, M. J. & Clarke, A. J. High throughput discovery and design of strong multicomponent metallic solid solutions. *Scientific reports* **8**, 1–10 (2018).

[10] Sohn, S. S. *et al.* Ultrastrong medium-entropy single-phase alloys designed via severe lattice distortion. *Advanced Materials* **31**, 1807142 (2019). URL https://onlinelibrary.wiley.com/doi/abs/10.1002/adma.201807142. https://onlinelibrary.wiley.com/doi/pdf/10.1002/adma.201807142.

[11] Li, Z., Pradeep, K. G., Deng, Y., Raabe, D. & Tasan, C. C. Metastable high-entropy dual-phase alloys overcome the strength–ductility trade-off. *Nature* **534**, 227–230 (2016). URL https://doi.org/10.1038/nature17981.
